# 

**Published:** 1897-07

**Authors:** 


### TO OUR SUBSCRIBERS.

SUBSCRIBERS to the JOURNAL will bear in mind that our rule of *all subscriptions in advance* has been rigidly enforced since the December, 1896, issue. All subscriptions terminate at the expiration of the period paid for. This rule applies to all. We always send notice of termination by a bill for the new year, and also a receipted acknowledgment for moneys received, and a reference to this or a postal card inquiry will inform you when your term expires. We have adopted this rule after the most mature consideration of every aspect of the situation and the most severe monetary losses in the history of the publication of the JOURNAL. This system has been adopted after some very unpleasant experiences with our fellow-veterinarians to whom credit has been extended for from one to five years, and we hope by this means to avoid a repetition of such unpleasantness in the future.

### PERSONAL.

Dr. N. S. Mayo, of Manhattan, Kas., still retains his connection with the Veterinary Department of the State Agricultural College.

Dr. D. McIntosh, of Champaign, Ill., has placed upon the market a book on the diseases of horses and cattle. It is intended, as the author says, for the layman, student, and young practitioner.

Dr. A. Smith, of Toronto, Can., is one of the creditors, to the extent of seven hundred dollars, of Charles Boyle, the turfman, who recently failed.

Editor Buckley, of the *Horseshoers' Journal*, has wrought remarkable changes in that publication, all in the best interests of the craft. The *Journal* is bright, fresh, and more readable than ever.

Veterinarian G. Howard Davison and bride will spend two months in Europe on their wedding tour.

Drs. Leonard Pearson, C. H. Magill, and Edward Kellner were the examining board for City Veterinarian of Philadelphia in June. In this examination experience and fitness counted equal to the results of the written practical scientific questions.

Dr. C. R. Jolly, of Atlanta, Ga., was a visitor to the JOURNAL office in June. Ten days were being spent among Northern cities and renewal of college ties.

Dr. H. W. Hawley, with a partner, conducts a large livery and sale stable at the Union Stock Yards, Chicago, Ill.

Drs. Wyatt Johnston and Paul Paquin were visitors to the Convention of the American Medical Association at Philadelphia in June.

Dr. Leonard Pearson was a welcomed visitor to the section on State Medicine of the American Medical Association at Philadelphia, having by vote been accorded the privilege of the floor in debate.

The list of applicants before the examining board of Philadelphia for the position of city veterinarian included Drs. Frank H. Nice, Walter L. Hart, acting city veterinarian; John Z. Tintsman, C. J. Marshall, W. H. Neff, J. Price Vasey, E. H. Moore, and Charles Bland.

Dr. George Coester, of Detroit, Mich., has succeeded, as State veterinarian, Dr. E. A. A. Grange, who has held this post for several years.

Dr. W. H. Dalrymple, of Baton Rouge, La., is among the editorial corps of the Southern Farmer Co., publishers of the *Southern Farmer*.

Dr. Ernest N. Hutchinson, of San Francisco, Cal., inspector under the Bureau of Animal Industry, has been detailed to Portland, Ore.

Dr. J. F. Reid, of Decatur, Ill., read recently an interesting paper on "Profitable Horse-breeding" before the Farmers' Institute in Decatur.

Veterinarian Wm. B. Werntz, of Philadelphia, has recently taken possession of a hotel property near the city, where he will devote his future labors as a landlord and public entertainer.

Dr. H. D. Hanson, of New York City, has been compelled to relinquish practice for a month and repair to the mountain regions of New York State for recuperation of his impaired health.

Prof. W. L. Williams, of Ithaca, N. Y., passed the examination of the New York State Board of Veterinary Medical Examiners in June. He thinks the examination questions in some branches are severe.

Dr. C. J. Sihler, who has been in charge of the government meat-inspection service at Kansas City for several years past, has been transferred to Tennessee and placed in charge of the government experiment station in Hickman county in that State, to eradicate hog-cholera and swine-plague.

Dr. S. E. Bennett, recently in charge of the United States meat-inspection at Milwaukee, succeeds Dr. Sihler at Kansas City.

Dr. Clarence Loveberry, of Columbus, Ohio, managing editor of the *Antiquarian*, has been appointed assistant-curator of the Ohio State Museum.

Dr. W. B. E. Miller, of Camden, N. J., has received an appointment under the Bureau of Animal Industry, and was ordered to New York City for duty on July 15th.

Veterinarians John T. Cunningham and L. Parker were the inspectors at the horse-show in June at Providence, R. I.

Dr. W. J. Waugh, of Jefferson Barracks, Mo., has been detailed to report at Fort Ethan Allen, Vt.

Dr. Bernard Gunther, of Brooklyn, was a visitor at Philadelphia, in June, attending the National Saengerfest as a member of the Arion Singing Society of Brooklyn. He also paid a visit to the JOURNAL office.

Dr. A. N. Lushington, graduate of the Veterinary Department of the University of Pennsylvania, a successful applicant for a license before the Pennsylvania State Board of Veterinary Medical Examiners, was an applicant also before the Virginia Board at Richmond, June 23d.

Dr. Wm. H. Harbaugh, of Richmond, Va., is reported in failing health.

Dr. W. S. Kooker, of Philadelphia, was seriously ill in June, but we are glad to report his convalescence.

Veterinarian Frank Allen, of Chicago, has been chosen a member of the Illinois Board of Horseshoers.

The many friends of Veterinarian S. D. Larzelere, of Jenkintown, Pa., will mingle their sincere sympathy with him in the great affliction that has come upon him in the loss of his wife.

Dr. J. McBirney has been placed in charge of the Bureau of Animal Industry experimental work in stamping out hog-cholera in Page county, Iowa.

Assistant State Veterinarians Joseph Hughes, J. F. Ryan, and A. H. Baker, of Chicago; A. S. Alexander, of Evanston; J. Stallman, of Pontiac; J. T. Reid, Decatur; H. G. Pyle, Springfield; R. C. Mylne, Aurora; and Geo. Ditewig, of East St. Louis, Ill., have resigned, owing to the selection by Governor Tanner of a non-graduate for veterinarian.

Veterinarians D. W. Patton, G. A. Johnson, and R. P. Steddon were the examiners of the horseshoers' students of anatomy and physiology at the Kansas City Veterinary College.

Dr. Thomas Fallon, of New York City, has been chosen second Vice-President of the Empire State organization of master-shoers.

Among the prominent veterinarians noted at the Philadelphia Horse Show were Drs. Alexander Glass, W. B. Montgomery, E. H. Magill, A. F. Schrieber, Leonard Pearson, and Charles M. Cullen, of Philadelphia; W. H. Ridge, Trevose; J. C. Bartholomew, of Berwyn; S. D. Larzelere, of Jenkintown; L. O. Lusson, of Ardmore, Pa.; F. H. Mackie, of Fairhill, Md.; and A. W. Clement, of Baltimore, Md.

Dr. H. D. Gill, of New York City, was one of the veterinarians at the recent horse show at Baltimore.

Dr. W. G. Benner, of Doylestown, Pa., had a very extended article upon tuberculosis in a recent issue of the *Daily Democrat*. Many of the ill-understood aims and purposes of the State Live-stock Sanitary Board were fully explained and commented upon.

Dr. Henry Vander Roest, of Newark N. J., is under arrest in that city, charged with manslaughter of Charles Furman, colored, whom he wounded with a pitchfork. Dr. Vander Roest claims to have been defending himself from an attack made on him by Furman owing to the non-payment of a small debt.

Dr. Wm. Herbert Lowe was re-elected to the office of City Veterinary Surgeon of Paterson, N. J.

Dr. John McBirney has been transferred from Chicago to Sioux City, Ia., and placed in charge of the government inspection at that point, succeeding Dr. A. B. Morse, who was transferred to Des Moines, Ia.

